# Effects of different rotation crops on soil physicochemical properties and microbial community structure in continuous cotton fields

**DOI:** 10.3389/fmicb.2026.1740768

**Published:** 2026-02-27

**Authors:** Yushui Duan, Hao Zhang, Chen He, Gang Gao, Qiuxiang Tang

**Affiliations:** Engineering Research Center for Cotton, Ministry of Education, College of Agriculture, Xinjiang Agricultural University, Urumqi, China

**Keywords:** cotton, functional prediction, rotation crop, soil microbiome, soil physicochemical properties

## Abstract

Cotton monoculture is widespread in the oasis cotton-growing region of Xinjiang. Long-term continuous cropping has led to declines in soil fertility and imbalances in microbial communities, constraining sustainable, green production. Crop rotation is an effective agronomic practice to mitigate the deleterious effects of continuous cropping; however, the selection of rotation crops and the regulatory mechanisms by which rotation reshapes the soil micro-ecology require systematic clarification. Using continuous cotton (CK) cropping as the control, we combined high-throughput amplicon sequencing with soil physicochemical analyses to evaluate the effects of four previous-crop schemes—cotton → peanut (CPC), cotton → soybean (CSC), cotton → rapeseed (CRC), and cotton → maize (CMC)—on soil properties and the microbial community structure. Relative to CK, the CPC, CSC, and CRC treatments led to significantly reductions in yield and gross output value ranging from 38.72 to 62.23% and 34.54 to 55.35%, respectively. Although the net profit under CPC treatment decreased by 36.27% relative to CK, the benefit–cost ratio showed no significant difference. CPC significantly increased soil organic matter, available phosphorus, NH4^+^–N, and NO3^−^–N, while decreasing the pH and electrical conductivity, demonstrating the best overall improvement in soil fertility. In the fungal community, under CPC, Basidiomycota and Mortierellomycota significantly increased by 17.15 and 52.37%, respectively, whereas Basidiomycota significantly increased under CSC and CRC (by 17.15 and 20.58%). Functional guild analysis indicated that all four rotation schemes significantly reduced the relative abundance of plant pathogen fungi, with the greatest decrease under CPC (36.80%), with statistically significant differences. In the bacterial community, CPC significantly increased Actinobacteriota, Gemmatimonadota, and Firmicutes by 16.20, 15.75, and 29.73%, respectively, while CRC rose substantially Bacteroidota by 28.58%. Bacterial metabolism constituted the major predicted functional category (79.27–79.68%), no significant differences between treatments. Redundancy analysis identified soil moisture and the N/P ratio as key drivers of the variation in the fungal community, while bacterial communities were regulated by N/P, pH, and organic matter. Overall, rotation alleviated continuous-cropping constraints by optimizing soil properties and the soil microbial structure. The results provide an empirical basis for improving soil microbiomes and designing sustainable planting strategies in oasis cotton systems.

## 1 Introduction

Cotton is the most important cash crop in Xinjiang. As of 2023, Xinjiang’s cotton planting area reached 2,369.3 thousand hectares, accounting for 84.98% of China’s total cotton acreage and producing 5.112 million tons—90.99% of the national output (National Bureau of Statistics, 2023). As China’s largest base for high-quality cotton, continuous monoculture is widespread in Xinjiang. However, prolonged continuous cotton (CK) cropping progressively degrades soil physicochemical properties, leading to nutrient imbalances, reduced microbial diversity, and an increased incidence of soil-borne pathogens, ultimately undermining yield formation (Gu et al., 2012). Crop rotation can optimize soil physicochemical attributes by introducing species from different botanical families (Li et al., 2023). In addition, crop rotation can balance nutrient utilization to avoid excessive depletion of specific elements (Delgado-Baquerizo et al., 2016) and restructure the soil microbial community by enriching beneficial taxa, thereby jointly mitigating the adverse effects associated with continuous cropping.

As the most active component of soil ecosystems, the structure and diversity of the soil microbial community are key indicators of the soil quality and health status (Kumar et al., 2017). Different rotation regimes exert varying influences on the microbial community structure and diversity; an appropriately designed rotation can maintain community diversity while enhancing microbial activity and function. Sun et al. (2020) and Wang et al. (2023) reported that a millet–maize rotation significantly increased the abundances of Mortierella and Glomeromycota among soil fungi, thereby promoting nutrient decomposition and cycling. Zhang Z. et al. (2025) and Zhang S. J. et al. (2025) found that a wheat–maize rotation significantly increased the relative abundance of Ascomycota, a type of fungus that is predominantly saprotrophic and capable of decomposing plant cellulose and other recalcitrant organic substrates. Yao et al. (2019) demonstrated that a peanut–watermelon–sweet potato rotation increased the abundances of Paenibacillus-like bacteria, Trichoderma, and Brevundimonas, enriching beneficial/antagonistic groups and thus lowering the risk of soil-borne diseases in peanuts. Jin et al. (2022) demonstrated that in a continuous winter-wheat–summer-maize annual rotation, introducing summer soybeans at different rotational intervals increased the relative abundance of potentially beneficial fungi. Zhang et al. (2024) reported that an alfalfa–triticale rotation had limited effects on bacterial community diversity but reduced fungal community richness, and that the result was potentially linked to changes in the soil nitrogen and phosphorus contents.

In Xinjiang, the constraints associated with CK cropping—such as soil nutrient imbalances, declines in soil microbial diversity, and reduced crop productivity—have become increasingly pronounced, forming a major bottleneck to the sustainable development of the regional cotton industry. Although prior investigations have verified crop rotation as an effective means to improve soils degraded by continuous monoculture, the range of crops examined remains limited, and studies involving rotation of crop groups widely cultivated in Xinjiang, such as legumes (Fabaceae), crucifers (Brassicaceae), and grasses (Poaceae), are lacking. Meanwhile, the soil microbial community is taxonomically and functionally complex, and the mechanisms by which rotation with different crops alters the microbial community dynamics in cotton systems remain poorly understood. Therefore, elucidating the changes in soil physicochemical properties and the microbial community under rotation between cotton and crops from different botanical families will provide essential information for supporting sustainable cotton production in the oasis agroecosystems of Xinjiang.

This study was conducted on an oasis field in Xinjiang that had been continuously planted with cotton for 30 years, aiming to address the following questions: (i) how the soil microbial community composition responds to different rotation schemes, and (ii) which soil parameters primarily drive changes in the microbial community composition under different rotation regimes.

## 2 Materials and methods

### 2.1 Experimental site

This experiment was conducted in 2023 at the Laolonghe Farm experimental field of the Western Center, Chinese Academy of Agricultural Sciences, in Changji, Xinjiang (31°24′97″N, 121°29′88″E), at an elevation of 600 m. The area has a typical continental arid climate, with an annual evaporation of 1,787 mm, an annual sunshine duration of 2,833 h, a mean annual air temperature of 6.6 °C, a mean annual precipitation of 160 mm, and a frost-free period of 160 days. The soil is classified as grey desert soil. The background soil physicochemical properties were as follows: pH 7.98; bulk density 1.48 g/cm^3^; soil organic matter (SOM) 12.61 g/kg; total N 0.65 g/kg; available P 22.04 mg/kg, and available K 296.00 mg/kg.

### 2.2 Field experimental design

Cotton was continuously grown on the site from 1992 to 2022; rotation trials commenced in 2023. A single-factor randomized block design was used. CK served as the control, and four rotation schemes were established: cotton → peanut (CPC), cotton → soybean (CSC), cotton → rapeseed (CRC), cotton → maize (CMC). The tested cultivars were as follows: cotton “Zhongmian 113;” peanut “Huayu 60;” soybean “Heinong 87;” rapeseed “Huayouza No. 62;” and maize “Xinyu 67.” The experimental area was 77 m in length and 34.65 m in width; each plot measured 172.50 m^2^. Three plastic mulch strips, each 2.05 meters wide, were laid within each plot. All plant and soil samples were collected from the middle strip to further minimize edge effects. Field management practices were consistent across treatments.

### 2.3 Soil sampling

After the harvest of each crop in 2023, soil samples were collected from the 0–20 cm layer of each plot using a five-point plum-blossom pattern. The samples were passed through a 1 mm sieve to remove plant and animal residue and homogenized. The fresh soil was kept refrigerated at a low temperature and promptly transported to the laboratory. One portion was stored at −80 °C for microbial analyses; one portion was stored at 4 °C for determination of soil water content and physicochemical properties, including NH_4_^+^–N and NO_3_^−^–N; and one portion was air-dried for measurements of pH, electrical conductivity (EC), SOM, and available phosphorus (AP), among other physicochemical indices.

### 2.4 Measured parameters and methods

#### 2.4.1 Crop yield measurement

##### 2.4.1.1 Cotton yield

After reaching the boll-opening stage, cotton within each plot was manually harvested and weighed to calculate seed cotton yield.

##### 2.4.1.2 Soybean yield

During the late maturity stage, soybeans from 2 rows within each plot were harvested, threshed, and weighed to estimate yield per hectare. Three sampling points were selected per plot, with 5 consecutive plants chosen at each point to measure pods per plant, seeds per plant, and 100-seed weight. These values were then used to calculate pod number and seed number per unit area.

##### 2.4.1.3 Peanut yield

At harvest, representative sample quadrats of 5.4 m^2^ were selected from each plot for yield determination.

##### 2.4.1.4 Rapeseed yield

In the rapeseed season, 6 representative rapeseed plants were randomly selected from each plot. Siliques per plant were counted, and 30 siliques were randomly chosen from each plant to determine seeds per silique. The average was used as the mean seeds per silique. Thousand-seed weight was measured by randomly selecting 6 sets of 1,000 fully air-dried seeds and weighing them using a seed counting board.

##### 2.4.1.5 Maize yield

At maturity, three sample sections were randomly selected per plot. Two rows of maize from each section were harvested for yield component analysis, including ear number per plot, yield, and grain 100-kernel weight. Theoretical yield was finally calculated based on a standard moisture content of 14%.

#### 2.4.2 Measurement of plant biomass, nitrogen and phosphorus uptake

##### 2.4.2.1 Dry matter accumulation

Plant samples were placed in kraft paper bags, deactivated at 105 °C for 30 min, and then dried at 80 °C to constant weight before weighing. Dry matter accumulation per plant and per population was calculated based on planting density.

##### 2.4.2.2 Nitrogen uptake (mg/plant)

Total plant nitrogen concentration was determined using the micro-Kjeldahl method after digestion with concentrated H_2_SO_4_–H_2_O_2_. Nitrogen uptake was calculated as nitrogen concentration multiplied by biomass.

##### 2.4.2.3 Phosphorus uptake (mg/plant)

Plants were washed and separated into aboveground and belowground parts, dried, weighed, and ground. Plant phosphorus concentration was determined using the molybdenum antimony anti-colorimetric method after H_2_SO_4_–H_2_O_2_ digestion. Plant phosphorus uptake was obtained by multiplying plant phosphorus concentration by plant dry weight.

#### 2.4.3 Determination of soil physicochemical properties

The soil water content was determined by the oven-drying method. The soil pH was measured using a pH meter. SOM was quantified by potassium dichromate oxidation–colorimetry. Alkali-hydrolyzable nitrogen was measured by the alkali diffusion method, ammonium N (NH_4_^+^–N) was determined by indophenol blue colorimetry, and nitrate N (NO_3_^−^–N) was determined by UV spectrophotometry. The content of AP was determined by the molybdenum–antimony anti-colorimetry method and measured by flame photometry. All methods followed the procedures in the reference soil agro-chemical analysis (Bao, 2000).

#### 2.4.4 Economic benefit calculation


Total input=Material costs+Labor costs;

$$\begin{array}{l} \text{Total revenue}=\text{Economic value of harvested}\;\\ \text{products}\;\mathrm{per}\;\text{hectare}=\text{Yield}\times \text{Crop price}; \end{array}$$

Netprofit=Total revenue–Total input;

Benefit–cost ratio=Total revenue÷Total input.

### 2.5 DNA extraction and high-throughput sequencing

Soil microbial samples were preserved in BIOSHARP 5 mL microbial cryovials. Total genomic DNA was extracted using the VAMNE Stool/Soil DNA Extraction Kit (Vazyme Biotech Co., Ltd., Nanjing, China; Cat. No. DM401-C3-P2). The V3–V4 hypervariable region of the bacterial 16S rRNA gene was amplified with primers 338F (5′-ACTCCTACGGGAGGCAGCA-3′) and 806R (5′-GGACTACHVGGGTWTCTAAT-3′), while the fungal ITS1 region was amplified with primers ITS1F (5′-CTTGGTCATTTAGAGGAAGTAA-3′) and ITS2 (5′-GCTGCGTTCTTCATCGATGC-3′). PCR amplicons were purified using Vazyme VAHTSTM DNA Clean Beads (Vazyme, Nanjing, China), quantified with the Quant-iT PicoGreen dsDNA Assay Kit (Invitrogen, United States), and subsequently sequenced on an Illumina HiSeq 2500 platform (2 × 300 bp paired-end) with three replicates per sample. Raw sequencing reads were subjected to quality filtering using Trimmomatic (version 0.33) (Bolger et al., 2014) and primer removal using Cutadapt (version 1.8.3) (Martin, 2011). To obtain both high-resolution sequence variants and enable comparison with traditional similarity-based methods, we employed two parallel bioinformatic pipelines: (1) a denoising-based approach using the DADA2 plugin within QIIME2 2020.6 to generate an amplicon sequence variant (ASV) table, and (2) a clustering-based approach using USEARCH (version 10) (Edgar, 2013) to cluster sequences into operational taxonomic units (OTUs) at a 97% similarity threshold, with chimera removal performed by UCHIME (Knight, 2011). All downstream analyses, including taxonomic annotation and diversity calculations, were performed based on the derived ASV and OTU tables. A total of 1,216,371 raw paired-end reads were obtained for the bacterial community across 15 samples, yielding 1,143,209 high-quality reads after processing. For the fungal community, 1,088,997 raw paired-end reads were obtained, resulting in 952,319 high-quality reads following the same preprocessing steps. Taxonomic annotation for bacterial OTUs was performed against the SILVA Release 132 database. Alpha-diversity indices (ACE, Chao1, Shannon, Simpson) were calculated using the core diversity analyses pipeline within QIIME2 2020.6.

### 2.6 Statistical analysis

Preliminary data sorting was performed using Excel 2021. Statistical analysis and one-way analysis of variance (ANOVA) were conducted using DPS 2021 software (Version 9.01) to assess differences in soil physicochemical properties, yield, economic benefits, and microbial characteristics among different treatments. Results in tables are presented as mean ± standard deviation, with the significance level set at *p* < 0.05. Subsequent data analysis and graphical visualization were performed using Origin 2021 software (Version 9.8.0.200) and R language. Within QIIME2, the ASV table was used to evaluate various alpha diversity indices, including the Chao1 index, ACE index, Shannon index, and Simpson index. Single-factor analysis of variance (ANOVA) was employed, with Tukey’s HSD and Duncan’s tests used to compare treatment means. Prior to conducting the ANOVA, Levene’s test was applied to assess the homogeneity of variances. R v3.1.1 (with the VennDiagram package v1.6.9) was used to analyze the number of shared and unique phyla of soil bacteria and fungi across all samples, visualized via Venn diagrams. Using the SILVA database as a reference, a naive Bayes classifier was employed for taxonomic annotation of feature sequences, obtaining species classification information for each feature. Community composition at each sample was then statistically analyzed across various levels (phylum, class, order, family, genus, species). QIIME software was used to generate species abundance tables at different taxonomic levels, which were then visualized using R language tools to create community structure plots at respective taxonomic levels, illustrating differences in bacterial and fungal community composition. The significance of these differences was tested using analysis of similarities (ANOSIM).

Distance-based redundancy analysis (RDA) was performed using R software to explore the relationships between soil physicochemical properties and soil bacterial and eukaryotic taxa. Linear discriminant analysis Effect Size (LEfSe) analysis was conducted to display differentially abundant features at the phylum, class, order, family, and genus levels, explaining differences among various soil sampling locations and intercropping treatments. LEfSe employed the non-parametric factorial Kruskal–Wallis sum-rank test to identify features with significantly different abundances (phylum, class, order, family, genus, species), followed by linear discriminant analysis (LDA) to estimate the effect size of each differentially abundant feature. Features were considered significantly different if they met the criteria of *p* < 0.05 and an effect size threshold (on a log10 scale) of 3.5. All bioinformatics analyses were completed using the online tools available on the BMK Cloud platform.

## 3 Results

### 3.1 The impact of different crop rotation patterns on crop yield and economic benefits

The yield and economic returns of different preceding crops varied according to the cultivation indices, as summarized in Table 1. Compared with the CK treatment, the four rotation treatments significant increased plant nitrogen and phosphorus uptake by 30.09–65.66% and 3.55–45.50%, respectively. In contrast, the CPC, CSC, and CRC treatments resulted in reductions in dry matter accumulation, yield, gross output value, and net profit relative to CK, with significant decreases ranging from 33.47–83.55%, 38.72–62.23%, 34.54–55.35%, and 36.27–66.58%, respectively. Conversely, the CMC treatment significant increased dry matter accumulation and yield by 82.55 and 31.58%, respectively. Additionally, although the net profit under the CPC treatment was 36.27% lower than that under CK, no significant difference was observed in the benefit–cost ratio.

**Table 1:** Yield and economic benefit of different crop rotations.

Planting pattern	DM (g/plant)	PNU (mg/plant)	PPU (mg/plant)	Yield (kg/hm^−2^)	GOV (CNY/hm^−2^)	NP (CNY/hm^−2^)	OIR
CK	51.32 ± 4.23b	1417.96 ± 71.29d	294.08 ± 10.02c	6143 ± 56.48b	39930 ± 113.46a	19830 ± 106.30a	1.98a
CPC	38.45 ± 3.74c	2218.57 ± 27.63b	304.91 ± 9.39bc	3764 ± 31.12c	26138 ± 518.31b	12638 ± 452.77b	1.95a
CSC	37.42 ± 4.08c	2028.40 ± 16.85c	315.44 ± 10.35b	3374 ± 23.90d	21859 ± 450.01d	8359 ± 224.95d	1.62c
CRC	27.96 ± 5.92d	1059.58 ± 81.90e	275.84 ± 12.01d	2320 ± 57.85e	17828 ± 426.89e	6628 ± 337.02e	1.59c
CMC	294.06 ± 17.31a	4128.94 ± 59.66a	539.56 ± 24.72a	8979 ± 40.32a	24063 ± 330.08c	9913 ± 232.48a	1.70b

Table values are expressed as mean ± standard deviation. Different lowercase letters following data in the same row indicate significant differences between treatments (*p* < 0.05). CK: continuous cotton; CPC: cotton → peanut; CSC: cotton → soybean; CRC: cotton → rapeseed; CMC: cotton → maize. DM, dry matter accumulation amount; PNU, plant nitrogen uptake; PPU, plant phosphorus uptake; GOV, gross output value; NP, net profit; OIR, output–input ratio.

### 3.2 The effects of different rotation schemes on soil physicochemical properties

Different rotation schemes exhibited variation in the effects on soil physicochemical properties (Table 2). Relative to the CK control, all four rotation modes increased SOM, AP, NH_4_^+^–N, and NO_3_^−^–N, while reducing the soil pH and EC. The CPC and CSC treatments produced significant increases compared to CK of 9.48–10.56% for SOM, 14.15–39.68% for AP, 5.33–17.61% for NH_4_^+^–N, and 18.10–28.89% for NO_3_^−^–N. In contrast, EC and pH decreased by 2.23–11.09% and 2.16–5.54%, respectively, but the differences were not statistically significant. In addition, under CPC, there were significantly increased in AP and NO_3_^−^–N by 11.84–28.09% and 4.31–18.87%, respectively, compared to the other rotation treatments.

**Table 2:** Soil physicochemical properties of cotton fields under different crop rotations.

Cropping pattern	Electrical conductivity (μS/cm)	Soil moisture content (%)	pH	Soil organic matter (g kg^−1^)	Available phosphorus (mg kg^−1^)	NH_4_^+^–N (mg kg^−1^)	NO₃^−^–N (mg kg^−1^)
CK	1271 ± 93.20a	12.56 ± 0.87a	7.88 ± 0.23a	12.02 ± 0.71c	20.49 ± 1.94c	4.69 ± 1.7c	73.38 ± 3.92c
CPC	1230 ± 57.15ab	13.68 ± 0.52a	7.68 ± 0.15a	13.16 ± 0.33ab	28.62 ± 2.61a	4.94 ± 0.97a	94.58 ± 3.94a
CSC	1130 ± 98.99c	13.87 ± 0.47a	7.71 ± 0.13a	13.41 ± 0.25a	24.54 ± 1.83b	5.56 ± 1.03a	90.50 ± 3.83a
CRC	1164 ± 73.64bc	13.25 ± 0.36a	7.76 ± 0.18a	12.78 ± 0.31bc	25.23 ± 2.32b	3.84 ± 1.02c	76.73 ± 4.27bc
CMC	1208 ± 76.98ab	13.02 ± 0.25a	7.78 ± 0.21a	12.12 ± 0.12c	20.58 ± 1.44c	4.51 ± 1.22b	84.14 ± 4.52b

Table values are expressed as mean ± standard deviation. Different lowercase letters following data in the same row indicate significant differences between treatments (*p* < 0.05). CK: continuous cotton; CPC: cotton → peanut; CSC: cotton → soybean; CRC: cotton → rapeseed; CMC: cotton → maize.

### 3.3 The effects of different rotation modes on OTU clustering of soil microorganisms

**Figure 1:**
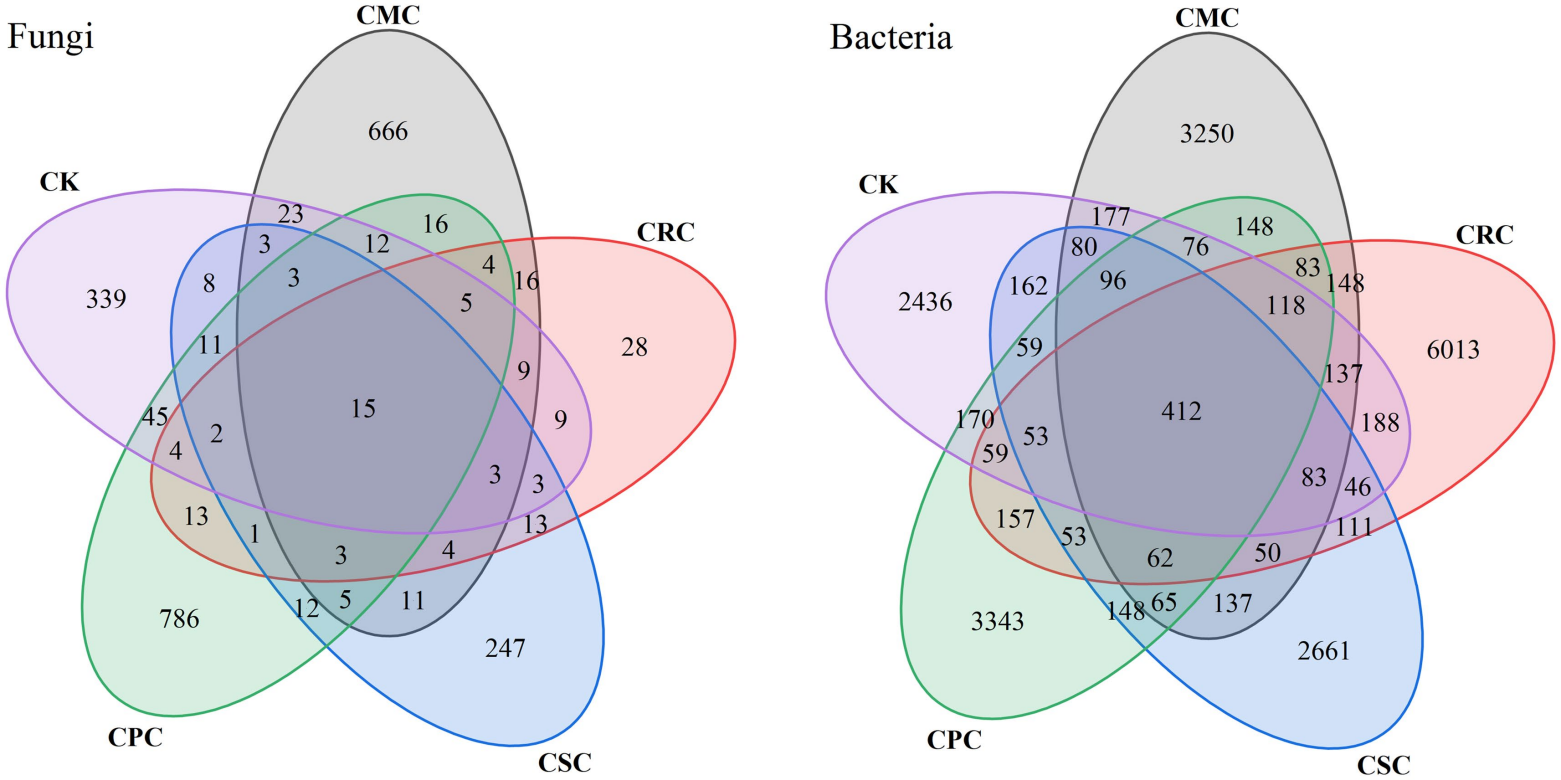
Venn diagram of fungal and bacterial OTU richness under different crop rotations. CK: continuous cotton; CPC: cotton → peanut; CSC: cotton → soybean; CRC: cotton → rapeseed; CMC: cotton → maize.

A total of 2,579 effective fungal OTUs were identified using a 97% sequence similarity threshold; the distribution patterns are shown in Figure 1, fungi. The numbers of fungal OTUs under the CK, CSC, CPC, CRC, and CMC treatments were 471, 821, 344, 937, and 392, respectively, indicating that different rotation schemes influenced the richness of fungal taxa. The analysis of shared and unique OTUs showed that only 15 fungal OTUs were common to all treatments, accounting for 0.58% of the total fungal OTUs, suggesting the presence of a small core fungal consortium across treatments. Concurrently, each rotation mode harbored treatment-specific OTUs: the numbers of unique fungal OTUs under CK, CSC, CPC, CRC, and CMC were 339, 666, 247, 786, and 288, respectively, representing 13.14, 25.82, 9.58, 30.48, and 11.17% of the total fungal OTUs.

At the same 97% similarity level, 20,710 effective bacterial OTUs were obtained (Figure 1, bacteria). The numbers of bacterial OTUs under CK, CSC, CPC, CRC, and CMC were 5,051, 4,292, 4,278, 5,102, and 7,702, respectively, demonstrating that rotation treatments also affected bacterial richness. An analysis of shared/unique taxa showed that 412 bacterial OTUs were common to all treatments, accounting for 1.99% of the total bacterial OTUs, again indicating a small core consortium across treatments. Treatment-specific bacterial OTUs were also evident: CK, CSC, CPC, CRC, and CMC harbored 3,250, 2,436, 2,661, 3,343, and 6,013 unique bacterial OTUs, corresponding to 15.69, 11.76, 12.85, 16.14, and 29.03% of the total bacterial OTUs, respectively.

### 3.4 Effects of different rotation schemes on microbial richness and diversity

In this study, four α-diversity indices—ACE, Chao1, Shannon, and Simpson—were calculated to evaluate the diversity characteristics of the soil microbial communities (Table 3). The results revealed significant differences in α-diversity among sample types. Compared to CK, all four crop rotation treatments significantly enhanced the α-diversity levels of both the soil bacterial and fungal communities (*p* < 0.05).

**Table 3:** Alpha diversity of soil fungi and bacteria under different crop rotations.

Microbial communities	Cropping pattern	ACE index	Chaol index	Shannon index	Simpson index
Fungi	CK	164.18 ± 11.48b	167.67 ± 15.17b	5.98 ± 0.52a	0.9800 ± 0.01a
	CPC	201.06 ± 13.25a	216.98 ± 21.31a	6.02 ± 0.64a	0.9600 ± 0.01a
	CSC	199.76 ± 5.48a	215.50 ± 15.40a	6.81 ± 0.75a	0.9700 ± 0.01a
	CRC	205.37 ± 10.04a	217.82 ± 19.84a	6.68 ± 0.72a	0.9600 ± 0.01a
	CMC	206.43 ± 11.58a	205.22 ± 11.78a	6.40 ± 0.41a	0.9800 ± 0.00a
Bacteria	CK	1645.90 ± 18.56c	1644.25 ± 19.07c	9.79 ± 0.19ab	0.9959 ± 0.00a
	CPC	2050.34 ± 61.75a	2073.48 ± 23.28a	10.04 ± 0.19a	0.9981 ± 0.00a
	CSC	2089.11 ± 22.31a	2085.75 ± 21.58a	9.83 ± 0.04ab	0.9979 ± 0.00a
	CRC	1733.74 ± 15.82b	1744.68 ± 8.82b	9.61 ± 0.08b	0.9983 ± 0.00a
	CMC	1771.47 ± 8.94b	1769.34 ± 14.22b	9.81 ± 0.03ab	0.9931 ± 0.00a

Table values are expressed as mean ± standard deviation. Different lowercase letters following data in the same row indicate significant differences between treatments (*p* < 0.05). CK: continuous cotton; CPC: cotton → peanut; CSC: cotton → soybean; CRC: cotton → rapeseed; CMC: cotton → maize.

In fungal communities, different crop rotation patterns significantly increased the ACE index and Chao1 index. The respective ACE, Chao1, and Shannon indices under the CPC, CSC, CRC, and CMC treatments were increased significantly by 17.81–20.47%, 18.30–23.02%, and 0.66–12.19% relative to the CK treatment. Notably, there were no significant differences in fungal α-diversity indices among the four rotation treatments (CPC, CSC, CRC, and CMC).

For the bacterial communities, different crop rotation patterns significantly increased the ACE index Chao1 index and Shannon index, the ACE and Chao1 indices under the CPC, CSC, CRC, and CMC treatments were significantly higher than those of the CK, with the increases ranging from 5.07 to 21.22% for ACE and 5.76 to 21.17% for the Chao1 index. In contrast, neither the Simpson index for the bacterial communities showed significant differences among treatments, suggesting that crop rotation primarily influenced bacterial richness (as reflected by the ACE and Chao1 indices) rather than community evenness (partially indicated by the Shannon and Simpson indices). Furthermore, among the four rotation treatments, CPC and CSC resulted in significantly higher ACE and Chao1 indices compared to CRC and CMC, indicating that the crop combinations in the CPC and CSC treatments may be more effective in enhancing soil bacterial richness.

### 3.5 The effects of rotation on the relative abundance of soil microorganisms

**Figure 2:**
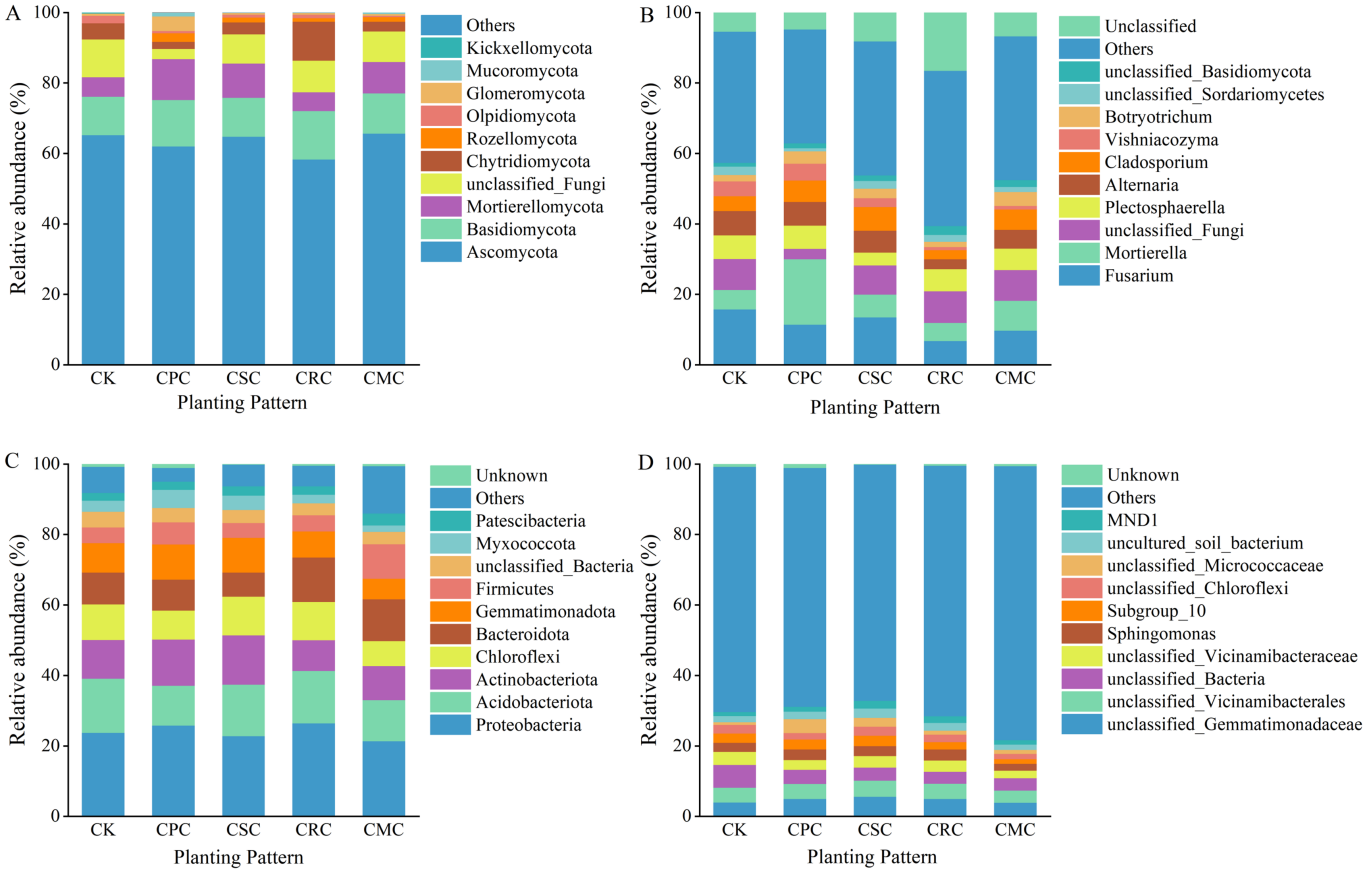
Changes in relative abundance of fungi at the phylum **(A)** and genus **(B)** levels, and bacteria at the phylum **(C)** and genus **(D)** levels under different crop rotations. CK: continuous cotton; CPC: cotton → peanut; CSC: cotton → soybean; CRC: cotton → rapeseed; CMC: cotton → maize.

Different crop rotation schemes significantly altered the fungal community composition, with notable disparities observed among the various treatments (Figure 2A). Across all treatments, the phylum Ascomycota accounted for 58.29–65.64% of the total fungal community, while Basidiomycota represented 10.92–13.75%. Compared to the CK treatment, the CPC rotation resulted in significant reductions in the relative abundances of Ascomycota, unclassified_Fungi, Olpidiomycota, and Chytridiomycota, by 4.89, 72.65, 72.14, and 56.48%, respectively. In contrast, the relative abundances of Basidiomycota and Mortierellomycota were significantly increased by 17.15 and 52.37%. Additionally, under the CMC treatment, the relative abundances of Basidiomycota and Mortierellomycota increased significantly by 4.55 and 37.84%, respectively, compared to CK. Significant differences in fungal community composition were also detected among treatments. At the genus level (Figure 2B), *Fusarium* accounted for 6.75–13.49%, *Mortierella* for 5.18–18.59%, and *Plectosphaerella* for 3.68–6.74% across treatments. Relative to CK, the CPC treatment significantly reduced the relative abundances of *Fusarium*, *Plectosphaerella*, and *Alternaria* by 27.62, 1.63, and 3.19%, respectively, while significantly increased the relative abundances of *Mortierella*, *Cladosporium*, *Vishniacozyma*, and *Botryotrichum* by 70.31, 31.16, 10.57, and 49.58%, respectively.

In contrast to the fungal community, the effects of crop rotation on the bacterial community structure were more pronounced (Figure 2C). Compared to CK, the CPC treatment significantly increased the relative abundances of Proteobacteria, Actinobacteriota, Gemmatimonadota, and Firmicutes by 7.84, 16.20, 15.75, and 29.73%, respectively, while reducing Acidobacteriota and Chloroflexi by 26.16 and 18.43%. In comparison, the CMC and CSC treatments decreased Proteobacteria by 10.06 and 4.08%, and Actinobacteriota by 24.07 and 2.80%, respectively. The CRC treatment increased Bacteroidota by 28.58% but reduced Gemmatimonadota by 12.14%. At the bacterial genus level (Figure 2D), *unclassified_Gemmatimonadaceae* and *unclassified_Vicinamibacterales* accounted for 3.88–5.60% and 3.45–4.61% across treatments, respectively. Compared to CK, the CPC treatment significantly increased the relative abundances of *unclassified_Gemmatimonadaceae* and *Sphingomonas* by 19.72 and 13.45%, respectively, while decreasing *unclassified_Bacteria* and *unclassified_Vicinamibacteraceae* by 37.36 and 25.81%. The CRC and CSC treatments alsosignificant increased *unclassified_Gemmatimonadaceae* by 19.23 and 58.75%, and *Sphingomonas* by 18.41 and 9.51%, respectively, while significant reduced *unclassified_Vicinamibacteraceae* by 14.21 and 12.37%.

### 3.6 RDA of soil properties and microbial communities under different rotations

**Figure 3:**
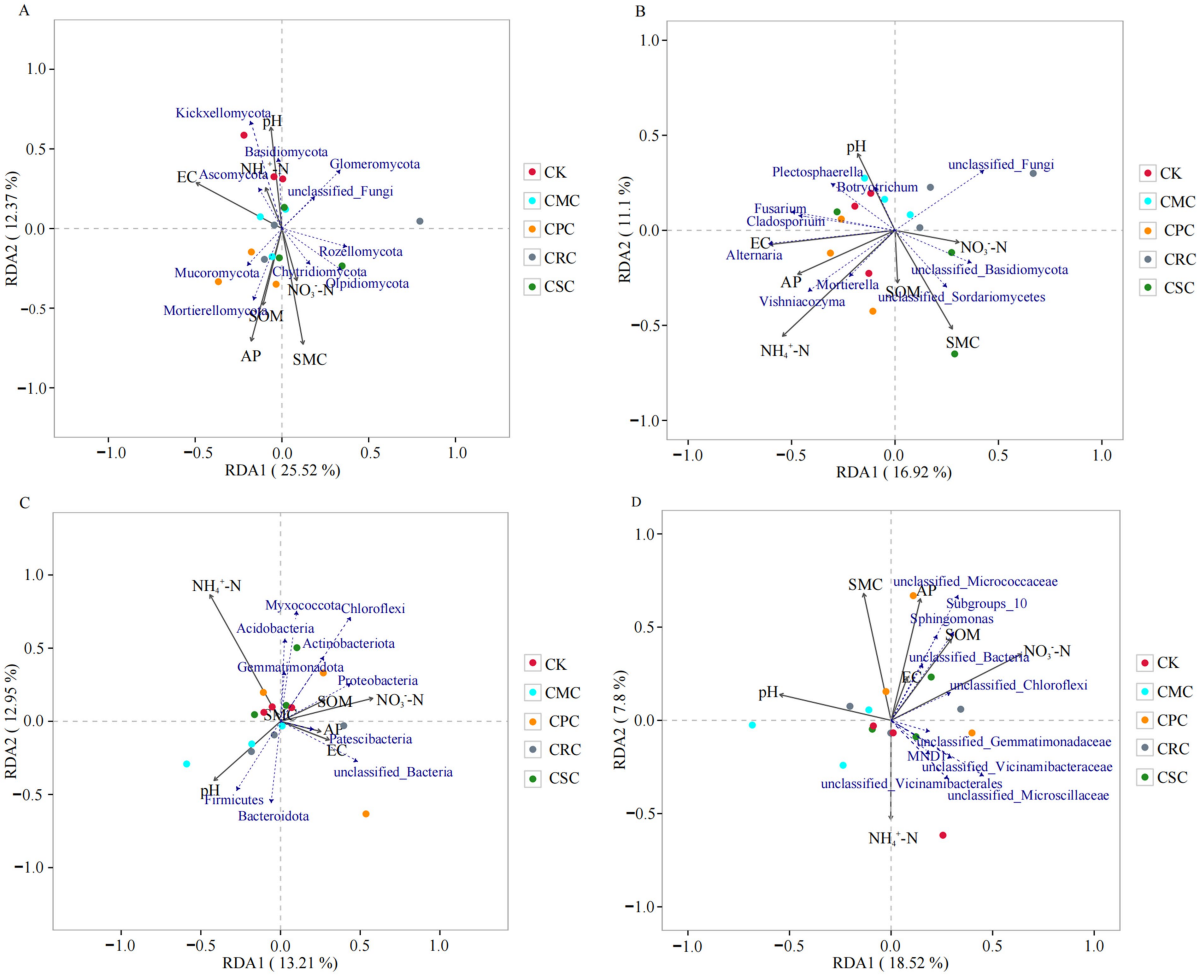
RDA analysis of soil physicochemical properties and fungal phylum level **(A)**, genus level **(B)**, bacterial phylum level **(C)**, and genus level **(D)** communities under different previous crops. CK: continuous cotton; CPC: cotton → peanut; CSC: cotton → soybean; CRC: cotton → rapeseed; CMC: cotton → maize.

Redundancy analysis (RDA) was performed to elucidate the relationships between soil fungal community diversity and environmental factors in cotton fields (Figures 3A,B). The results indicated that the divergence in fungal community structure under different crop rotation treatments was significantly correlated with soil physicochemical properties.

At the phylum level, the RDA ordination explained 25.52 and 12.37% of the total variation in fungal community composition along the first and second axes, respectively (Figure 3A), suggesting that the selected soil properties effectively accounted for the structural differences in fungal communities. Monte Carlo permutation tests further identified EC, AP, pH, soil moisture content (SMC), and SOM as key environmental drivers of the variation in the fungal community structure (*p* < 0.05). Specifically, SOM, SMC, and AP exhibited significant positive correlations with Ascomycota. SOM and SMC showed significant positive correlations with Mortierellomycota, Rozellomycota, and Mucoromycota. EC demonstrated significant negative correlations with Mortierellomycota and Rozellomycota. NH₄^+^–N showed significant negative correlations with unclassified_Fungi and Chytridiomycota. NH₄^+^–N and NO₃^−^–N exhibited significant positive correlations with Mortierellomycota and Rozellomycota. At the genus level, the RDA axes explained 16.92 and 11.1% of the variation in fungal community composition, respectively (Figure 3B). Soil AP was significantly positively correlated with the relative abundances of *Mortierella* and *Vishniacozyma*, and NH_4_^+^–N was positively correlated with *Alternaria* and *Mouterella*. In contrast, SMC was negatively correlated with *Fusarium* and *Alternaria*.

The ordination analysis also revealed distinct associations between soil bacterial community diversity and physicochemical factors at both the phylum and genus levels (Figures 3C,D). At the phylum level, the RDA axes explained 13.21 and 12.95% of the bacterial community variation (Figure 3C), indicating that the bacterial community structure was influenced by the combined effects of multiple soil properties. Different factors exhibited varying influences on bacterial phyla: NH₄^+^–N showed a significant negative correlation with Actinobacteriota and Chloroflexi, while exhibiting a significant positive correlation with Acidobacteria. SOM demonstrated significant positive correlations with Proteobacteria, Gemmatimonadota, and Bacteroidota. pH was negatively correlated with Acidobacteria, Proteobacteria, Bacteroidota, and Gemmatimonadota. EC showed significant negative correlations with Actinobacteriota, Bacteroidota, and Gemmatimonadota. SMC was positively correlated with Actinobacteriota and Acidobacteria, and displayed a significant positive correlation with Proteobacteria. At the genus level, the RDA axes accounted for 18.52 and 7.8% of the bacterial community variation, respectively (Figure 3D). AP was positively correlated with *Sphingomonas* and *Subgroup_10*, while SOM was positively correlated with *unclassified_Bacteria* and *unclassified_Chloroflexi.* Conversely, NH_4_^+^–N and pH were negatively correlated with *unclassified_Microscillaceae*.

### 3.7 The significant impact of different crop rotations on the enrichment of soil microorganisms

**Figure 4:**
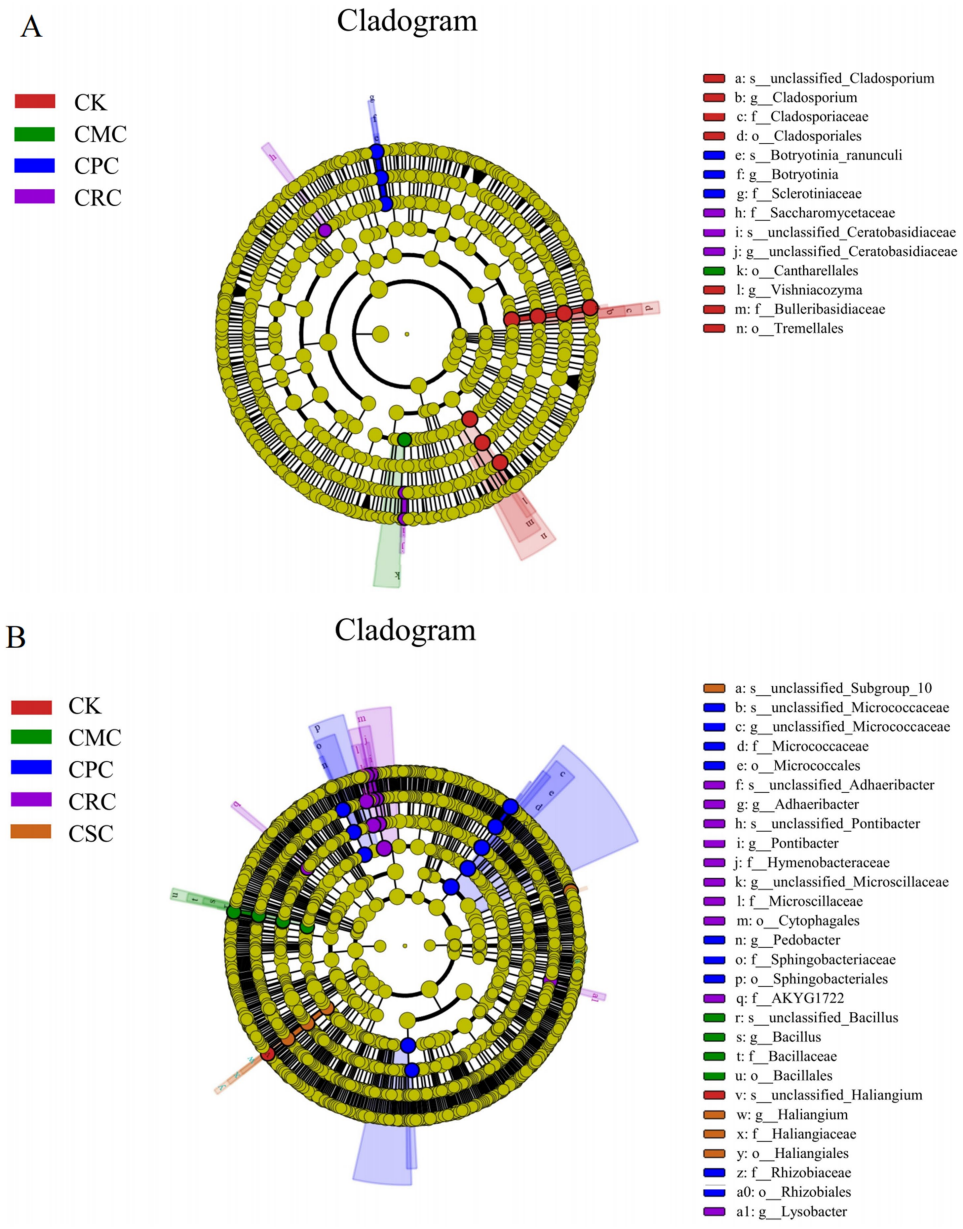
LEfSe analysis of microorganisms under different crop rotations: **(A)** branching diagram of LEfSe analysis for fungi; **(B)** CK: continuous cotton; CPC: cotton → peanut; CSC: cotton → soybean; CRC: cotton → rapeseed; CMC: cotton → maize.

The LEfSe analysis revealed distinct soil microbial taxa associated with different crop rotation treatments. A total of 14 differentially abundant taxa were identified in the fungal community (Figure 4A). Specifically, *s_unclassified Cladosporium*, *Cladosporium* (genus), Cladosporiaceae (family), Cladosporiales (order), *Vishniacozyma*, f_Bulleribasidiaceae, and o_Tremellales were significantly enriched in the CK treatment. The order Cantharellales was enriched under the CMC treatment. In the CPC treatment, *s_Botryotinia_ranunculi*, *Botryotinia* (genus), and Sclerotiniaceae (family) were significantly enriched. Meanwhile, Saccharomycetaceae (family), *s_unclassified_Ceratobasidiaceae*, and *g_unclassified_Ceratobasidiaceae* were markedly enriched in the CRC treatment.

LEfSe analysis revealed that 28 different microbial taxa were identified in soil bacterial communities under different rotation treatments (Figure 4B). The species *s_unclassified_Haliangium* was significantly enriched in the CK treatment. Under the CPC treatment, multiple beneficial bacterial taxa were enriched, including Bacillaceae (family), *Bacillus* (genus), *s_unclassified_Bacillus*, Micrococcaceae (family), Micrococcales (order), *Pontibacter*, *s_classified_Micrococcaceae*, Sphingobacteriaceae, Sphingobacteriales, Rhizobiales, and Rhizobiaceae. The CRC treatment led to significant enrichment of *s_unclassified_Adhaeribacter*, *Adhaeribacter* (genus), *s_unclassified_Pontibacter*, Hymenobacteraceae, *Pontibacter,* Micrococcales *s_unclassified_Microscillaceae*, Micrococcaceae, Cytophagales, Rhizobiales, and *Lysobacter*. In the CSC treatment, the significantly enriched taxa included *s_unclassified_Subgroup_10*, *Haliangium* (genus), Haliangiaceae (family), and Haliangiales (order).

### 3.8 Predicted microbial functions under different rotations

**Figure 5:**
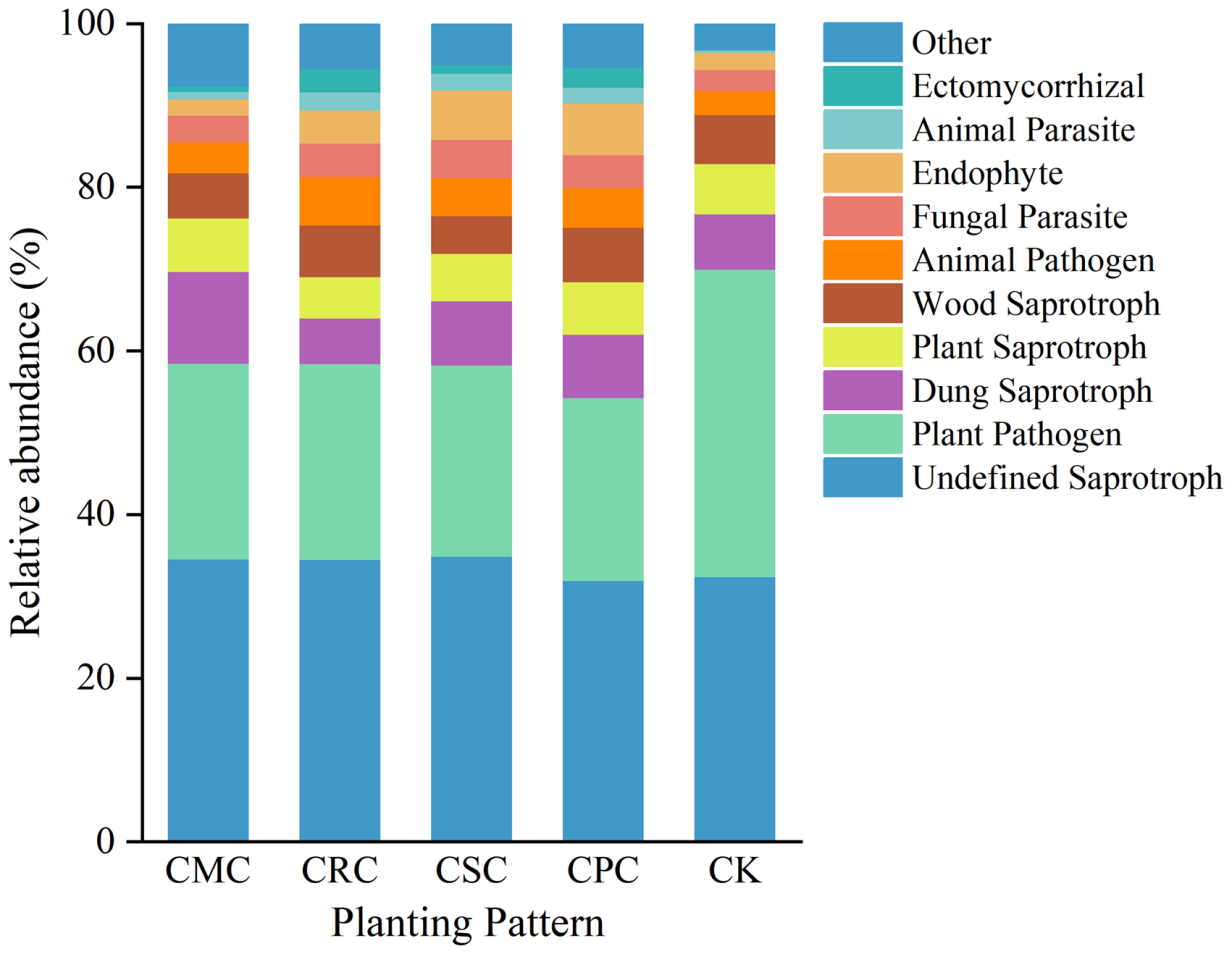
Predicted fungal functions under different crop rotations. CK: continuous cotton; CPC: cotton → peanut; CSC: cotton → soybean; CRC: cotton → rapeseed; CMC: cotton → maize.

FUNGuild was used to classify fungal trophic modes as pathotrophs (34.59–54.73%), symbiotrophs (6.31–10.19%), and saprotrophs (37.76–59.10%). These were further subdivided into undefined saprotrophs, plant pathogens, coprophilous saprotrophs, plant saprotrophs, wood saprotrophs, animal pathogens, fungal parasites, endophytes, animal parasites, ectomycorrhizal fungi, and others (Figure 5). Relative to CK, plant pathogens significantly declined by 40.40, 37.80, 36.25, and 36.36% under CPC, CSC, CRC, and CMC, respectively; endophytes significantly increased under CPC and CSC (6.26 and 6.04%; +66.45% and +65.23%), and coprophilous saprotrophs significantly increased under CPC, CSC, and CMC by 12.19%, 13.65%, and 39.55%. Undefined saprotrophs did not differ significantly among treatments. The relative abundance of plant pathogenic fungi significantly decreased under different crop rotation patterns, and the cotton → peanut treatment had a more pronounced effect on the predicted functional potential of soil fungi.

**Figure 6:**
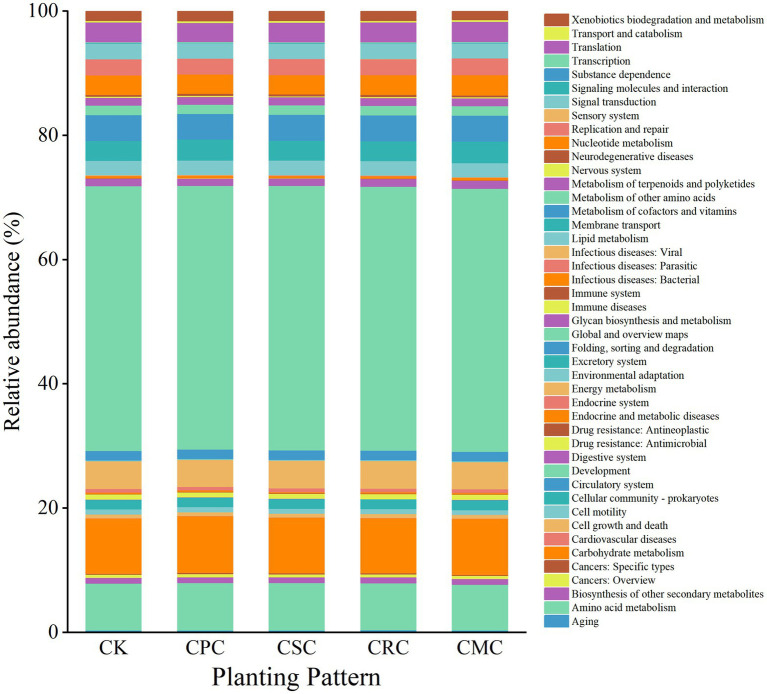
Column chart of soil bacterial secondary functions under different crop rotations. CK: continuous cotton; CPC: cotton → peanut; CSC: cotton → soybean; CRC: cotton → rapeseed; CMC: cotton → maize.

PICRUSt predictions based on the KEGG database showed Level 1 functions of soil bacteria were categorized into six major groups: metabolism, genetic information processing, environmental information processing, cellular processes, organismal systems, and human diseases. These were further subdivided into specific subfunctions (Figure 6). At Level-2, 45 subfunctions were detected; the main categories were global and overview maps (42.35–42.61%), carbohydrate metabolism (8.86–9.15%), amino acid metabolism (7.34–7.60%), energy metabolism (4.35–4.44%), membrane transport (3.21–3.46%), metabolism of cofactors and vitamins (4.10–4.20%), and nucleotide metabolism (3.12–3.30%). Compared to CK, CPC significantly decreased global and overview maps (−0.42%), energy metabolism (−1.80%), and nucleotide metabolism (−1.37%), while significantly increased amino acid metabolism (+1.05%), carbohydrate metabolism (+2.95%), and membrane transport (+5.31%). Under CMC, amino acid metabolism and global and overview maps were significantly declined (−2.39% and −0.61%), whereas carbohydrate metabolism and membrane transport were increased (+1.77% and +7.22%).

### 3.9 Analysis of soil physicochemical properties, microbial communities, and crop yield in cotton fields under different rotation systems

**Figure 7:**
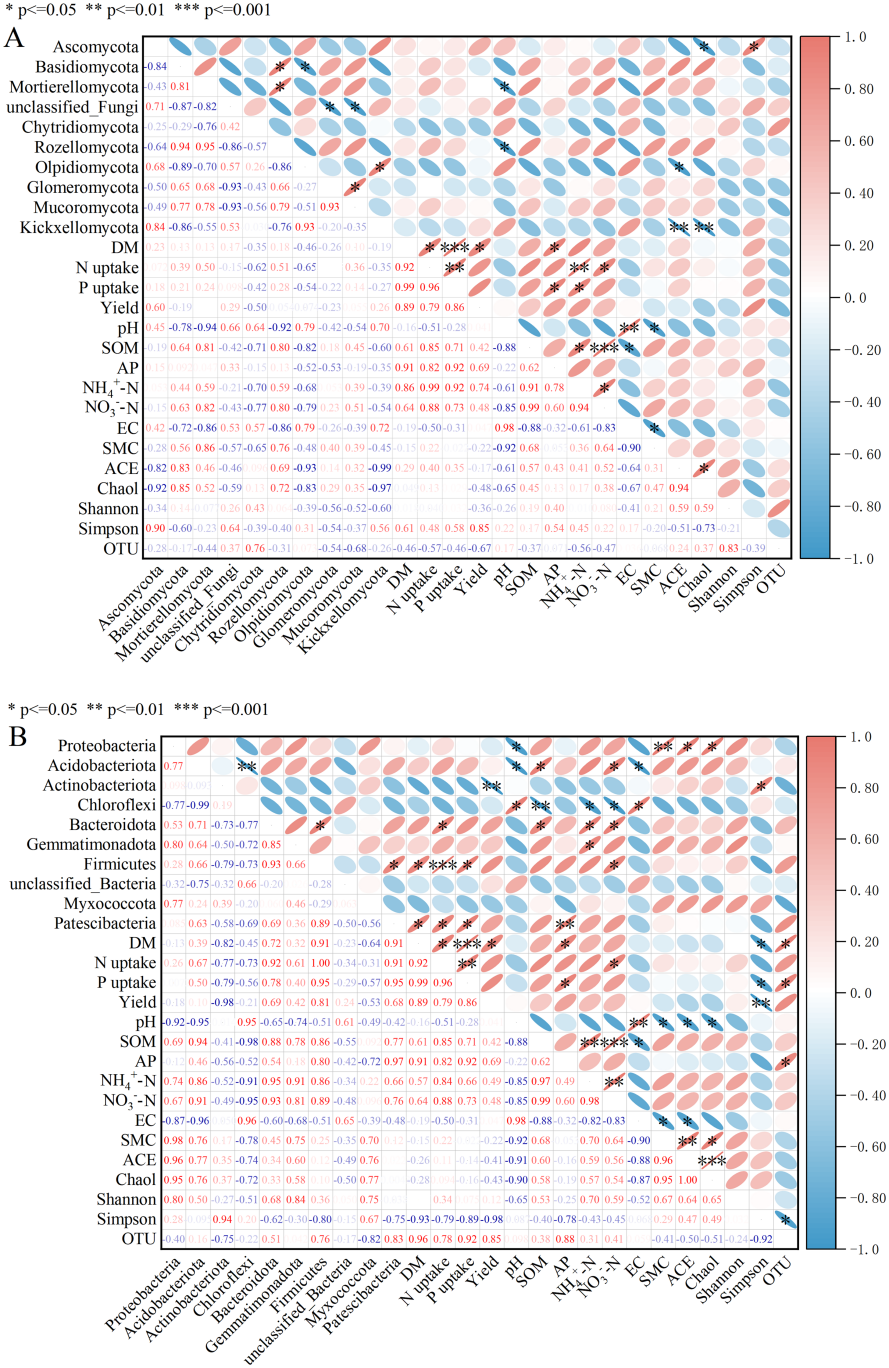
Correlation heatmap of soil physicochemical properties, microbial community, and crop yield in cotton fields under different crop rotations. CK: continuous cotton; CPC: cotton → peanut; CSC: cotton → soybean; CRC: cotton → rapeseed; CMC: cotton → corn. DM: dry matter accumulation; N uptake: plant nitrogen uptake; P uptake: plant phosphorus uptake; EC: electrical conductivity; SMC: soil moisture content; SOM: soil organic matter; AP: available phosphorus; ACE: ACE index; Chao1: Chao1 index; Shannon: Shannon index; Simpson: Simpson index; OTU: Operational taxonomic unit.

The coupling relationships among DM, Yield, N uptake, P uptake, soil microbial communities, and physicochemical factors were systematically evaluated using Pearson correlation analysis (Figures 7A,B). At the fungal level (Figure 7A), DM, Yield, N uptake, and P uptake showed correlation coefficients greater than 0.8 among themselves, indicating significant positive correlations and a high degree of synergy among dry matter accumulation, yield, and nitrogen and phosphorus uptake processes. At the dominant phylum level of the fungal community, DM, N uptake, and P uptake exhibited significant positive correlations with Ascomycota, Basidiomycota, Mortierellomycota, and Rozellomycota. Yield showed significant positive correlations with Ascomycota, unclassified_Fungi, and Kickxellomycota. In contrast, DM, Yield, N uptake, and P uptake were significantly negatively correlated with Chytridiomycota and Olpidiomycota. Furthermore, DM, Yield, N uptake, and P uptake were significantly positively correlated with SOM, AP, NH₄^+^–N, and NO₃^−^–N, but negatively correlated with EC. Meanwhile, these four indices generally showed significant positive correlations with fungal diversity indices such as ACE, Chao1, and Shannon, but were mostly negatively correlated with the Simpson index.

At the bacterial level (Figure 7B), DM, Yield, N uptake, and P uptake demonstrated significant positive correlations with the relative abundances of several dominant bacterial phyla, such as Proteobacteria, Actinobacteriota, Bacteroidota, and Firmicutes, while showing significant negative correlations with Actinobacteriota and Chloroflexi. Notably, N uptake and P uptake were more responsive to changes in these bacterial taxa. Regarding physicochemical properties, the four indices generally showed significant positive correlations with indicators such as SOM, AP, NH₄^+^–N, NO₃^−^–N, EC, and SMC, but were significantly negatively correlated with pH Simultaneously, they exhibited significant positive correlations with bacterial community diversity indices like ACE, Chao1, and Shannon, while showing negative correlations with the Simpson index.

## 4 Discussion

### 4.1 The effects of different rotation schemes on soil physicochemical properties

Continuous cropping is especially common in modern, intensive cotton production in Xinjiang; however, long-term monoculture produces a suite of soil ecological problems—including nutrient imbalances, deterioration of the physical structure, and declines in water-use efficiency—that severely constrain the sustainability of cotton agroecosystems (Lupwayi et al., 2024). In contrast, crop rotation can be tailored to plant-specific biological traits to purposefully adjust the soil physicochemical conditions and promote the balanced utilization of soil nutrients and water; thus, rotation constitutes an effective agronomic measure to alleviate the drawbacks associated with continuous cropping. Han S. et al. (2024) demonstrated in continuously cropped cotton fields of northern Xinjiang that rotation with wheat, soybean, maize, and processing tomato significantly improved the soil physicochemical properties, providing practical guidance for optimizing rotation systems in cotton fields.

The present study confirms that the ameliorative effects of different rotation schemes on the physicochemical properties of cotton-field soil vary significantly depending on the crop choice. Notably, rotation with peanut and soybean (both legumes) produced the most pronounced improvements: relative to long-term CK, the peanut rotation significantly increased the contents of AP, NH_4_^+^–N, and SOM by 28.41%, 23.20%, and 8.66%, respectively; the soybean rotation significantly reduced EC and pH by 11.45 and 2.16%, respectively, while significantly increasing NH_4_^+^–N, NO_3_^−^–N, and AP by 27.66%, 12.79%, and 16.50%, respectively. The results concur with the findings of Zhang et al. (2019), Zheng et al. (2021), Zhang Z. et al. (2025), and Zhang S. J. et al. (2025). Collectively, the results reinforce the recognized advantages of legumes within rotation systems: through symbiosis with rhizobia, legumes can meet approximately 40–60% of their nitrogen demand via biological nitrogen fixation, thereby substantially reducing the reliance on native soil N; moreover, decomposition of legume residues returns nitrogen to the soil, markedly elevating soil N levels.

It is noteworthy that the rotation effects were crop-specific. Under CRC rotation, the soil nitrogen content did not differ significantly from CK, consistent with the study of Tang et al. (2020); the core reason is that rapeseed is a typically high-N-demand crop whose growth cycle entails substantial nitrogen consumption, making net N accumulation in soil difficult to achieve (Ren and Lu, 2016). Under CMC rotation, the soil moisture content did not differ significantly from CK, in line with the study by Chai et al. (2022). This is possibly because maize and cotton are both high-water-use crops with similar temporal patterns of soil water demand, which limits the capacity of rotation to enhance the coordination and retention of soil moisture.

In sum, the extent to which rotation improves soil physicochemical properties in cotton fields depends on the choice of the rotation crop. Legumes (peanuts, soybeans) possess clear advantages for enhancing soil fertility (N, P, and SOM) and optimizing the chemical milieu (reductions in EC and pH), whereas rapeseed and maize have more limited effects on specific physicochemical indicators (i.e., N and water).

Rational cropping practices that leverage the advantages of rotation systems facilitate more efficient utilization of soil nutrient resources by crops. Studies have shown that after continuous cultivation of wheat and rapeseed for over three years, introducing legume crops into short-term rotations can enhance soil N and P availability and promote nitrogen recovery efficiency (NRE) and phosphorus recovery efficiency (PRE) (Song et al., 2021). Research by Han F. et al. (2024) indicated that maize/peanut rotation significantly improved the land equivalent ratio of subsequent intercropping and monoculture systems, leading to substantially higher economic benefits compared to continuous cropping. Tewolde et al. (2021) demonstrated that a cotton–maize–soybean rotation system increased both crop yield and the magnitude of soil mineral nutrient removal at harvest. Similarly, Zhou et al. (2021) reported that rotating soybean and maize with cotton enhanced crop yield, improved soil health, and provided additional ecosystem services and profitability.

### 4.2 Effects of different rotation schemes on the soil microbial community

As the most active functional component of soil ecosystems, the structure and diversity of the soil microbial community constitute core biological indicators for evaluating soil quality (Yan et al., 2024). By altering the physicochemical properties of the tilled layer and introducing crop-specific microbial consortia, rotation can markedly regulate the fungal abundance, diversity, and community composition (Hartman et al., 2020), thereby influencing the soil ecological functions. In the present study, compared to CK, the four types of crop rotation treatments can significantly increase the ACE index, Chao1 index, and Shannon index of fungi in the 0–20 cm topsoil layer. This aligns with the findings of Yang et al. (2022) in similar cotton-rotation systems and confirms the generality of rotation-induced enhancements in fungal richness and diversity. Mechanistically, rotation can optimize the soil C/N ratio and promote the decomposition and transformation of SOM, thereby providing more abundant carbon and nitrogen sources for fungal growth and indirectly enhancing fungal diversity and richness (Song et al., 2022). This process reflects a coordinated response between the soil physicochemical milieu and the microbial community.

At the phylum level, Ascomycota and Basidiomycota were the dominant fungal phyla across all treatments, with relative abundances of 58.29–65.64% and 10.92–13.75%, respectively. These results are consistent with previous studies in cotton and other agricultural ecosystems (Liu et al., 2021; Liu et al., 2018). As typical saprotrophic fungi, Ascomycota and Basidiomycota possess the ability to degrade lignin and decompose organic matter (Zhang et al., 2013), making these fungi key drivers of soil carbon cycling and nutrient transformation. The relative abundance of Mortierellomycota under CPC and CSC treatments significantly increased by 52.37 and 43.44% compared to CK, respectively. Fungi in the Mortierellomycota promote plant growth by facilitating the release of soil nitrogen and phosphorus and increasing soil organic carbon content (Ning et al., 2022).

Notably, the soil fungal diversity indices under the CPC and CSC treatments (legume rotation) were significantly higher than those under other schemes. This is likely related to the biological characteristics of leguminous crops: their symbiotic nitrogen fixation with rhizobia improves the soil nitrogen status, while their root exudates provide diverse ecological niches for fungi, thereby enhancing fungal community diversity (Liu H. et al., 2019; Liu J. X. et al., 2019).

At the genus level, compared to continuous cropping, the relative abundance of *Mortierella* was significantly increased by 70.25%, 14.26%, and 34.79% under the CPC, CSC, and CMC treatments, this can enhance soil fertility and strengthen plant uptake of nitrogen and phosphorus by degrading cellulose and inhibiting the growth of pathogenic fungi (Xiong et al., 2017). Concurrently, all rotation treatments reduced the relative abundance of *Fusarium*. This result directly demonstrates the important role of crop rotation in controlling soil-borne diseases and ensuring sustainable crop production (Hong et al., 2025).

RDA further revealed the driving effects of soil physicochemical factors on the fungal community structure, with SOM, SMC, and AP showed significant positive correlations with Ascomycota, while SOM and SMC were significantly positively correlated with Mortierellomycota, Rozellomycota, and Mucoromycota. In contrast, EC exhibited significant negative correlations with Mortierellomycota and Rozellomycota. NH_4_^+^–N was significantly positively correlated with *Mortierella* and *Alternaria*. These findings were consistent with those of Zhu et al. (2023) and Hao et al. (2024) indicated that the soil water content is also a key factor affecting fungal diversity, suggesting that changes in fungal community structure result from the interactive effects of multiple environmental factors rather than a single factor.

In summary, this study has confirmed that different rotation patterns can significantly regulate the fungal community structure, enhance fungal richness and diversity, and specifically alter the relative abundance of key fungal taxa (e.g., *Mortierella*, *Fusarium*) by modifying the soil physicochemical environment in cotton fields, thereby optimizing soil ecological function.

Compared to the fungal communities, soil bacterial communities are more sensitive to environmental change (Tang et al., 2023), and dynamic shifts in their structure and diversity can rapidly reflect changes in the quality of soil ecosystems. The alpha diversity analysis revealed that compared to CK cropping, the effects of different rotation treatments on soil bacterial community richness and diversity varied significantly. The four rotation treatments significantly increased the ACE index by 5.07–21.22% and the Chao1 index by 5.76–21.17%. These results were consistent with those of Wang et al. (2022), further confirming that the choice of rotation crop species is a key factor regulating bacterial community characteristics.

The regulatory effects of different rotation treatments on the dominant phyla showed distinct specificity and were closely linked to changes in soil nutrients. The CPC rotation increased the relative abundances of Proteobacteria, Actinobacteriota, and Gemmatimonadota by 7.84%, 16.20%, and 15.75%, respectively, while significantly decreasing the relative abundance of Chloroflexi by 18.43%. The results are similar to those reported by Nan et al. (2021) and Yin et al. (2020). The core mechanism lies in the ability of leguminous crops to significantly increase SOM, AP, and inorganic nitrogen. Proteobacteria and Actinobacteriota are typical “high-fertility-preferring” bacterial groups (Jiang et al., 2021), and their increased abundance exhibits a synergistic response to improvements in soil nutrient levels. Chloroflexi are typical oligotrophic bacteria whose growth and reproduction are sensitive to soil nutrient concentrations.

At the bacterial genus level, the CPC rotation increased the relative abundances of *unclassified_Gemmatimonadaceae* and *Sphingomonas* by 19.72 and 13.45%, respectively, consistent with the findings of Wang et al. (2020). *Sphingomonas* can exert probiotic functions by secreting plant growth-promoting substances and inhibiting pathogenic microorganisms, while *unclassified_Gemmatimonadaceae* participates in the degradation and transformation of SOM (Daniel et al., 2009). The increased abundances of these genera can synergistically affect soil ecological functions and create a favorable environment for crop growth.

The redundancy analysis identified the driving mechanisms of soil physicochemical factors on the bacterial community structure, with the regulatory effects of each factor showing clear group specificity: soil SOM showed significant positive correlations with Proteobacteria, Gemmatimonadota, and Bacteroidota, while pH exhibited significant negative correlations with Acidobacteria, Proteobacteria, Bacteroidota, and Gemmatimonadota indicating that a moderate decrease in soil pH is more conducive to the growth and reproduction of bacteria in these two phyla. This result aligns with the study by Shen et al. (2015), and the underlying mechanism is that pH indirectly regulates bacterial metabolic activity by altering the forms of nutrients and enzyme activities in the soil micro-environment. SOM was significantly positively correlated with Proteobacteria and *unclassified_Bacteria*, consistent with previous research (Li et al., 2016). Mechanistically, Proteobacteria possess a strong capacity for decomposing and utilizing organic matter, and the positive correlation between their abundance and SOM content essentially reflects the fact that SOM provides sufficient carbon and energy sources for this bacterial group (Fierer et al., 2007).

In summary, crop rotation improves soil ecological functions and crop yield by regulating the physicochemical environment of cotton fields, enhancing fungal and bacterial community richness and diversity, specifically optimizing functional microbial groups (e.g., Mortierella and Actinobacteria), and inhibiting pathogenic microorganisms. However, this study did not clarify interspecific interactions among microbial taxa, and the dynamic legacy effects of rotation soils require further investigation. Future research integrating high-throughput sequencing and molecular interaction experiments will provide more comprehensive support for the precise optimization of rotation systems.

This study represents a short-term rotational cropping experiment spanning only one year. Soil samples were collected exclusively from the 0–20 cm plow layer, failing to elucidate microbial interactions in deeper soil horizons or fully reveal the mechanisms by which crop rotation influences the critical root zone of cotton. Furthermore, functional predictions have not yet been validated using enzyme activity or metabolomics data; such validation will be conducted in subsequent experiments. Future studies will collect samples from the 0–40 cm profile and deeper layers. Integrating high-throughput sequencing with molecular interaction experiments will provide more comprehensive support for the precise optimization of crop rotation systems.

### 4.3 The effects of different rotation species on microbial ecological functions

The ecological functions of soil fungi are directly linked to crop growth, development, and health status. The composition and dynamic changes of fungal functional groups serve as key indicators for assessing the service capacity of soil ecosystems. In this study, the FUNGuild tool was utilized to predict micro-ecological functions, categorizing and predicting the ecological roles of soil fungi in cotton fields under various crop rotation treatments based on their resource utilization strategies and nutrient acquisition modes (Nguyen et al., 2016). This approach clarified the regulatory effect of rotation patterns on the structure of fungal functional groups, providing functional-level insights into the mechanisms by which crop rotation alleviates the problems associated with continuous cropping. Regarding pathotrophic fungi, their relative abundance in the CK cropping treatment was as high as 54.73%, while all rotation treatments significantly reduced their relative abundance by 30.61–36.80%, consistent with findings of Wang et al. (2010) in soils under continuous cropping systems. As a potential reservoir of plant pathogens, an excessively high abundance of pathotrophic fungi increases the risk of soil-borne diseases and negatively affects plant growth (Anthony et al., 2017). The results of this study demonstrated a significant increase in the relative abundance of *Fusarium* under continuous cropping. This genus comprises multiple pathogenic fungi that can secrete toxins (e.g., fumonisins and fusaric acid), which disrupt root cell structures, inhibit enzyme activity, and consequently impede plant growth and development (Chen et al., 2024). The suppressive effect of crop rotation on pathotrophic fungi essentially operates by altering the soil micro-environment and fungal ecological niches, thereby reducing the competitive advantage and colonization capacity of pathogens and mitigating their potential harm. This represents one of the key functional mechanisms through which crop rotation alleviates the negative effects of continuous cropping.

All rotation treatments significantly increased the relative abundances of saprotrophic fungi compared to the control treatment. The underlying mechanisms can be interpreted from two perspectives. On the one hand, saprotrophic fungi possess the ability to decompose complex organic matter, converting insoluble organic substrates into small-molecule nutrients accessible to plants (Nan et al., 2023). The differential input of crop residues during rotation provides diverse carbon and energy sources for saprotrophic fungi, accelerating the decomposition and transformation of SOM and thereby driving the enrichment of this fungal group. On the other hand, rotation modifies soil physicochemical properties, optimizing the micro-environment for saprotrophic fungal growth (Liu et al., 2014). The increased abundance of saprotrophic fungi not only enhances the soil’s nutrient supply capacity but also improves its structure through the decomposition of organic matter.

Symbiotic fungi play essential ecological roles in promoting nutrient uptake, enhancing stress resistance, and improving crop quality (Igiehon and Babalola, 2017). The results of this study showed that, compared to CK, CPC, CSC, and CRC rotation significantly increased the relative abundance of symbiotic fungi by 11.96%, 23.99%, and 26.30%, respectively. In contrast, CMC rotation did not exhibit a significant effect. This suggests that the enrichment of symbiotic fungi is strongly dependent on the specific crop species used in the rotation.

In summary, different rotation treatments optimize soil ecological functions by suppressing pathotrophic fungi, enriching saprotrophic fungi involved in the decomposition of organic matter, and promoting symbiotic fungi that enhance plant growth. This process constitutes a core mechanism through which crop rotation alleviates the negative effects of continuous cropping and improves soil quality.

Changes in the soil bacterial community structure are often accompanied by dynamic adjustments to their ecological functions, with differences in functional gene abundance serving as the molecular basis for these functional shifts. In this study, PICRUSt functional prediction analysis was employed to compare high-throughput sequencing data of bacterial communities with the KEGG database, systematically elucidating the regulatory effects of different rotation treatments on functional gene profiles and ecological functions of soil bacteria in cotton fields. This provides molecular-level evidence for understanding the mechanisms by which crop rotation improves soil ecosystem services.

At the level of primary functional classification, metabolism was the absolutely dominant function across all treatments, with a relative abundance of 79.28–79.69%. This result aligns with previous studies (Ding et al., 2021; Xia et al., 2020), confirming that metabolism is the core process through which soil bacteria participate in material cycling and energy transformation. The result indicates that bacteria convert organic and inorganic substances in the soil into forms utilizable by both themselves and crop plants, thereby linking soil fertility levels to crop growth requirements.

The regulatory effects of different rotation treatments on key metabolic functions of bacteria varied significantly and were closely related to the crop species used in the rotation. The CPC rotation treatment exhibited the most pronounced functional enhancement, increasing the abundance of metabolic functional genes by 0.20% compared to CK. Notably, the abundances of functional genes related to amino acid metabolism, carbohydrate metabolism, and membrane transport increased by 1.05%, 2.95%, and 5.31%, respectively. CSC and CRC rotations primarily enhanced the abundance of amino acid metabolism-related functional genes, with respective increases of 1.05 and 0.53%. The enhancement of these key metabolic functional gene abundances essentially reflects the directional succession of bacterial community functions driven by changes in the soil micro-environment induced by crop rotation. On the one hand, root exudates and residue decomposition products from different rotation crops provide diverse substrate resources for bacteria, stimulating the expression and enrichment of functional genes associated with substrate degradation and absorption (Chen et al., 2025). On the other hand, the improved soil nutrient status resulting from rotation further optimizes the bacterial growth environment, enhancing their metabolic activity and thereby promoting the abundance of functional genes directly involved in nutrient transformation, such as those involved in amino acid metabolism (He et al., 2013).

In this study, the relative abundances of bacterial functional categories related to metabolism, amino acid metabolism, and carbohydrate metabolism were significantly higher under different rotation treatments than in the control treatment, a pattern consistent with the aforementioned mechanism.

In conclusion, different crop rotation treatments can directionally enhance key metabolic functions related to nutrient transformation and material cycling (e.g., amino acid metabolism, carbohydrate metabolism) by regulating the functional gene profiles of soil bacteria in cotton fields, with the CPC rotation showing the most significant functional optimization effect. These results not only reveal the bacterial functional mechanisms through which crop rotation improves soil fertility and promotes crop growth but also provide functional-level evidence for the precise selection of crops in cotton rotation systems. Both FUNGuild and PICRUSt2 are prediction tools that require subsequent validation through measurements of microbial carbon and nitrogen content and enzyme activity (such as urease and phosphatase).

## 5 Conclusion

Compared to CK, all rotation regimes significantly increased soil water content, SOM, AP, and NO_3_^−^–N by 3.75–8.19%, 0.82–10.37%, 0.44–28.41%, and 4.37–22.41%, respectively; notably, the CPC rotation markedly enhanced soil C- and N-related nutrients by 28.41 and 22.41%, respectively. Fungal and bacterial α-diversity indices were significantly higher under all rotations than under continuous cropping. Relative to the control, Glomeromycota increased by 3.5% under the CPC rotation, and Mucoromycota increased by 6.07 and 4.24% under CPC and CSC systems, respectively. On the bacterial side, Proteobacteria and Actinobacteriota increased by 2.02 and 2.13% under the CPC rotation, indicating notable shifts in the dominant functional microbial groups of cotton soils. RDA identified soil pH, SMC, AP, and SOM as the key environmental drivers structuring the microbial communities. Fungal functional prediction identified pathotrophs as the most abundant group under continuous cropping, whereas rotation significantly enriched saprotrophs. Bacterial functional prediction identified metabolism as the dominant function across treatments; under the CPC rotation, the relative abundances of genes related to overall metabolism, amino acid metabolism, carbohydrate metabolism, and membrane transport were comparatively higher. Correlation heatmap analysis revealed that DM, Yield, N uptake, and P uptake were positively correlated with Ascomycota but negatively correlated with Chytridiomycota and Olpidiomycota. They also showed positive correlations with several dominant bacterial phyla, including Proteobacteria, Actinobacteriota, Bacteroidota, and Firmicutes, as well as generally exhibited positive correlations with physicochemical indicators such as SOM, AP, NH₄^+^–N, NO₃^−^–N, EC, and SMC. These results suggest that shifts in microbial communities under different crop rotation systems can influence soil nutrient supply capacity, thereby exerting potential effects on crop nutrient uptake and yield formation.

Taken together, the results indicate that rotation optimizes cotton soil ecosystems by improving soil physicochemical properties and reshaping the microbial community structure and function, with the CPC scheme exhibiting the greatest overall benefits. This work provides quantitative evidence with which to guide the selection of cotton rotation strategies and offers a theoretical basis for enhancing soil productivity and alleviating the problems associated with continuous cropping through microbial community regulation, thereby supporting sustainable agricultural development in cotton-growing regions. A long-term positioning experiment is currently underway, integrating soil enzyme activity measurements, microbial biomass analysis, and metabolomics to validate functional prediction results and decipher multidimensional driving factors. Simultaneously, we fully recognize that the long-term sustainability effects of crop rotation and its impacts on deeper soil layers require further investigation.

## Data Availability

The raw data supporting the conclusions of this article will be made available by the authors, without undue reservation.
